# Carnitine palmitoyltransferase 1C contributes to progressive cellular senescence

**DOI:** 10.18632/aging.103033

**Published:** 2020-04-14

**Authors:** Yongtao Wang, Tao Yu, Yanying Zhou, Shike Wang, Xunian Zhou, Limin Wang, Tianmiao Ou, Yixin Chen, Yawen Zhou, Huizhen Zhang, Ying Wang, Xiaomei Fan, Pan Chen, Frank J. Gonzalez, Aiming Yu, Peng Huang, Min Huang, Huichang Bi

**Affiliations:** 1 School of Pharmaceutical Sciences, Sun Yat-sen University, Guangzhou 510006, P.R. China; 2 Laboratory of Metabolism, Center for Cancer Research, National Cancer Institute, NIH, Bethesda, MD 20892, USA; 3 Laboratory of Human Carcinogenesis, Center for Cancer Research, National Cancer Institute, NIH, Bethesda, MD 20892, USA; 4 Department of Biochemistry and Molecular Medicine, Comprehensive Cancer Center, UC Davis School of Medicine, Sacramento, CA 95817, USA; 5 State Key Laboratory of Oncology in South China, Sun Yat-Sen University Cancer Center, Guangzhou 510275, P.R. China

**Keywords:** carnitine palmitoyltransferase 1C, stable transfection, senescence, mitochondria, metabolic reprogramming

## Abstract

Stable transfection manipulation with antibiotic selection and passaging induces progressive cellular senescence phenotypes. However, the underlying mechanisms remain poorly understood. This study demonstrated that stable transfection of the empty vector induced PANC-1 cells into cellular senescence. Metabolomics revealed several acylcarnitines and their upstream regulatory gene, carnitine palmitoyltransferase 1C (CPT1C) involved in fatty acid β-oxidation in mitochondria, were strikingly decreased in senescent PANC-1 cells. Low CPT1C expression triggered mitochondrial dysfunction, inhibited telomere elongation, impaired cell survival under metabolic stress, and hindered the malignance and tumorigenesis of senescent cells. On the contrary, mitochondrial activity was restored by CPT1C gain-of-function in senescent vector PANC-1 cells. PPARα and TP53/CDKN1A, crucial signaling components in cellular senescence, were downregulated in senescent PANC-1 cells. This study identifies CPT1C as a key regulator of stable transfection-induced progressive PANC-1 cell senescence that inhibits mitochondrial function-associated metabolic reprogramming. These findings confirm the need to identify cell culture alterations after stable transfection, particularly when cells are used for metabolomics and mitochondria-associated studies, and suggest inhibition of CPT1C could be a promising target to intervene pancreatic tumorigenesis.

## INTRODUCTION

A commonly used stable transfection manipulation for an exogenous gene with a selectable marker that remains in the genome of eukaryotic cells and their daughter cells is highly desirable [1]. Cells then remain alive and can be further cultivated under selective stress with passaging. However, stable transfection manipulation induces progressive cellular senescence phenotypes, leading to a common problem in cell culture. The underlying mechanisms of stable transfection-induced cellular senescence remain poorly understood. As reported in our previous study [2], extended passaging of PANC-1 cells were triggered into a replicative senescence process with low CPT1C levels. Low CPT1C expression further caused mitochondria dysfunction-associated metabolic reprogramming and impaired malignancy in senescent replicative PANC-1 cells. More importantly, knockdown of CPT1C in cancer cells induced mitochondrial dysfunction, senescence-like growth suppression and cellular senescence and further suppressed malignancy and tumorigenesis *in vivo* and xenograft tumor growth *in situ*. On the contrary, the gain-of-function of CPT1C reversed PANC-1 cell senescence and enhanced mitochondrial function. CPT1C was hence confirmed as a novel biomarker for mitochondrial dysfunction-associated cellular senescence [2]. Recently, microRNA-1291 and its mimic empty vector pCMV stably transfected PANC-1 cell lines were established to reveal the role of microRNA-1291 in pancreatic carcinoma cell metabolism and suppressed tumorigenesis [3]. It was found that stable transfection manipulation induces progressive PANC-1 cell senescence, but whether the underlying mechanisms of this senescence are mitochondrial dysfunction-associated or CPT1C-depent remain unclear. Therefore, the current study aimed to study the metabolomics change in stable transfection-induced progressive PANC-1 cell senescence and to reveal the underlying molecular signals involved in this progress. The metabolomics results demonstrate several acylcarnitines and their upstream regulatory gene, CPT1C, were strikingly decreased in senescent PANC-1 cells. CPT1C was further identified as a crucial regulator in stable transfection-induced PANC-1 cell senescence by inhibiting mitochondrial function-associated metabolic reprogramming. These findings suggest the need to identify cell culture alterations after stable transfection, particularly when cells are used for metabolomics and mitochondria-associated studies, and suggest inhibition of CPT1C could be a promising target to intervene pancreatic tumorigenesis.

## RESULTS

### Stable transfection-induced PANC-1 cell senescence

In our previous study, microRNA-1291 and its mimic empty vector pCMV stably transfected PANC-1 cell lines were established to reveal the role of microRNA-1291 in pancreatic carcinoma cell metabolism and suppressed tumorigenesis [3]. Here, it was confirmed that stable transfection of the empty vector pCMV in human pancreatic epithelioid carcinoma PANC-1 cells led to severe growth arrest and cellular senescence.

**Figure 1:**
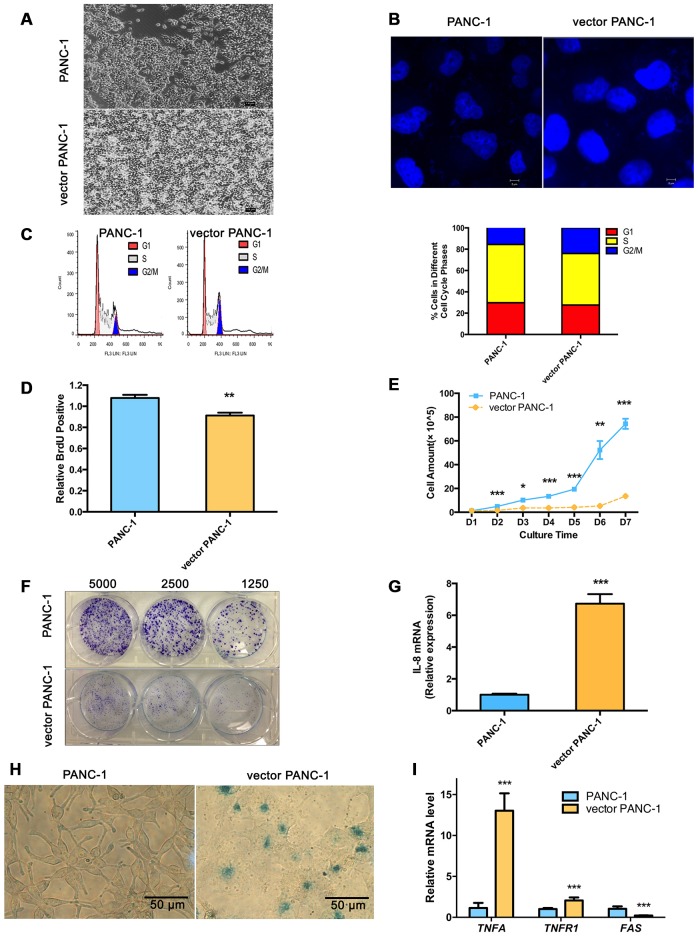
**Stable transfection-induced PANC-1 cell senescence.** (**A**) Morphology graph of vector PANC-1 cells. (**B**) Confocal fluorescent graph of the nuclei (blue fluorescence) morphology of vector PANC-1 cells. (**C**) An increased percentage of vector PANC-1 cells was arrested in G2/M phase. Graphic (top) and percentage (bottom) representations of cell cycle distributions are shown. This experiment was repeated independently three times. (**D**) Decreased BrdU incorporation during DNA synthesis in vector PANC-1 cells. Data are presented as the mean ± S.E.M, n = 4 (**p < 0.01). (**E**) Cell growth curve shows decreased proliferation of vector PANC-1 cells. Data are presented as the mean ± S.E.M, n = 3 (*p < 0.05, **p < 0.01, ***p < 0.001). (**F**) Decreased ability of vector PANC-1 cells to form colonies when seeded at the indicated dilutions. (**G**) Quantitative RT-PCR analysis of the upregulated key SASP factor, *IL-8* mRNA, in vector PANC-1 cells. Data are presented as the mean ± S.E.M, n = 3 (***p < 0.001). (**H**) SA-β-gal staining and positive senescence signal of vector PANC-1 cells. This experiment was repeated independently three times. (**I**) Activation of extrinsic apoptosis pathways was analyzed. See also Supplementary Figures 1 and 2.

Vector PANC-1 cells (PANC-1 cells stably transfected with the empty vector pCMV were characterized by an enlarged and flattened appearance arranged like flagstones with increased granularities in the cytoplasm (Figure 1A) compared with mock PANC-1 cells (untreated PANC-1 cells). Consistent with this result, degenerative changes of enlarged nuclei were also observed in vector PANC-1 cells (Figure 1B). To examine cell growth suppression of the senescence-associated phenotypes, flow cytometry was performed to determine whether stable transfection of the vector caused an increase in the population of vector PANC-1 cells in G2/M phase (Figure 1C). Vector PANC-1 cells exhibited lower proliferation than mock PANC-1 cells, as revealed by BrdU incorporation measured during DNA synthesis (Figure 1D) and cell growth curve tracing (Figure 1E). Furthermore, a weaker ability of vector PANC-1 cells to form cell colonies was observed (Figure 1F). *IL-8*, a key SA secretory phenotype (SASP) factor involved in the senescence process [4–7], was increased in vector PANC-1 cells compared to mock PANC-1 cells (Figure 1G). *IL-8* was negatively correlated (but without statistical significance) with *CPT1C* mRNA expression in pancreatic cancer patients (Supplementary Figure 2A), further supporting enhanced SASP in low-CPT1C-induced senescent vector PANC-1 cells. More importantly, β-galactosidase (SA-β-gal) staining showed that mock PANC-1 cells were nearly negative for β-gal, while vector PANC-1 cells were positive for senescent signals (Figure 1H). The mRNA levels of *TNF-α* and its receptor *TNFR1*, were significantly increased in vector PANC-1, indicating the activation of extrinsic apoptosis pathways in the senescent cells. However, *FAS* mRNA expression was reduced in the senescent cells, which might result from the negative feedback regulation of activation of TNF-α-TNFR1 pathway (Figure 1I).

Taken together, these data indicate that stable transfection of the empty vector triggered PANC-1 cells into a strong senescence-like growth suppression and severe cellular senescence.

### Metabolomics reveals a lower level of acylcarnitines in senescent vector PANC-1 cells, which is linked to reduced CPT1C expression

**Figure 2:**
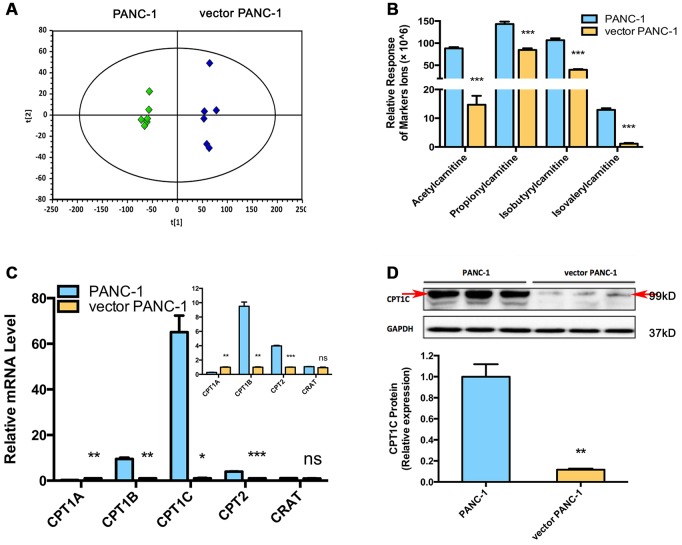
**Metabolomics reveals a lower level of acylcarnitines in senescent vector PANC-1 cells, which is linked to reduced CPT1C expression.** (**A**) PCA score plots of HILIC-ESI^+^-MS metabolomics profiles obtained from HILIC-ESI^+^-MS, n = 6/group. (**B**) Analysis of the relative response of acylcarnitine ions in senescent vector PANC-1 cells. Data are presented as the mean ± S.E.M, n = 6 (***p < 0.001). (**C**) Quantitative RT-PCR analysis of genes related to acylcarnitines. Data are presented as the mean ± S.E.M, n = 3 (ns indicates no significance, *p < 0.05, **p < 0.01, ***p < 0.001). The specific human primers to amplify corresponding mRNA were obtained from website of http://pga.mgh.harvard.edu/primerbank/ and PrimerDepot, and commercially available (Invitrogen) and shown in Supplementary Table 1. (**D**) Images and densitometric analysis of CPT1C protein bands of senescent vector PANC-1 cells. Data are presented as the mean ± S.E.M, n = 3 (**p < 0.01). See also Supplementary Figure 3.

Metabolomics analysis was performed to further identify potential regulators or biomarkers underlying cellular senescence induced by stable transfection of the empty vector pCMV. To identify the general trends in an unbiased way, unsupervised principal component analysis (PCA) was performed to reveal differences between the mock and vector PANC-1 cells. PCA scatter diagrams obtained from HILIC-ESI^+^-MS (Figure 2A) and HILIC-ESI^—^MS (Supplementary Figure 3A) showed a clear separation between the mock and vector PANC-1 cells, suggesting a distinct discrimination in the metabolome profiles between these two groups. S-plot of OPLS/DA models resulting from HILIC-ESI^+^- MS indicated four significantly changed ions (Supplementary Figure 3B). The ions were further specifically identified as acetylcarnitine (Supplementary Figure 3C), propionylcarnitine (Supplementary Figure 3D), isobutyrylcarnitine (Supplementary Figure 3E) and isovalerylcarnitine (Supplementary Figure 3F). Interestingly, the relative response of all of the marker ions was significantly reduced in senescent vector PANC-1 cells (Figure 2B).

To identify the potential drivers behind the dramatic decrease in acylcarnitine levels in senescent vector PANC-1 cells, the mRNA expression of genes involved in acylcarnitine transport was further determined. Specifically, *CPT1B* and *CPT2* mRNA levels were significantly decreased in vector PANC-1 cells, while *CPT1A* mRNA levels showed a slight increase and carnitine O-acetyltransferase (*CRAT*) mRNA expression remained unchanged (Figure 2C). However, *CPT1C* mRNA levels were the most strikingly decreased in vector PANC-1 cells compared to mock PANC-1 cells (Figure 2C). Furthermore, CPT1C protein levels were significantly reduced in senescent vector PANC-1 cells (Figure 2D). Together, these data imply that the decrease in CPT1C is the most important contributor to the transport of decreased acylcarnitines and may represent a driver of vector PANC-1 senescence.

### Dysfunctional mitochondria and inhibited telomere elongation in low-CPT1C-expressing senescent vector PANC-1 cells

**Figure 3:**
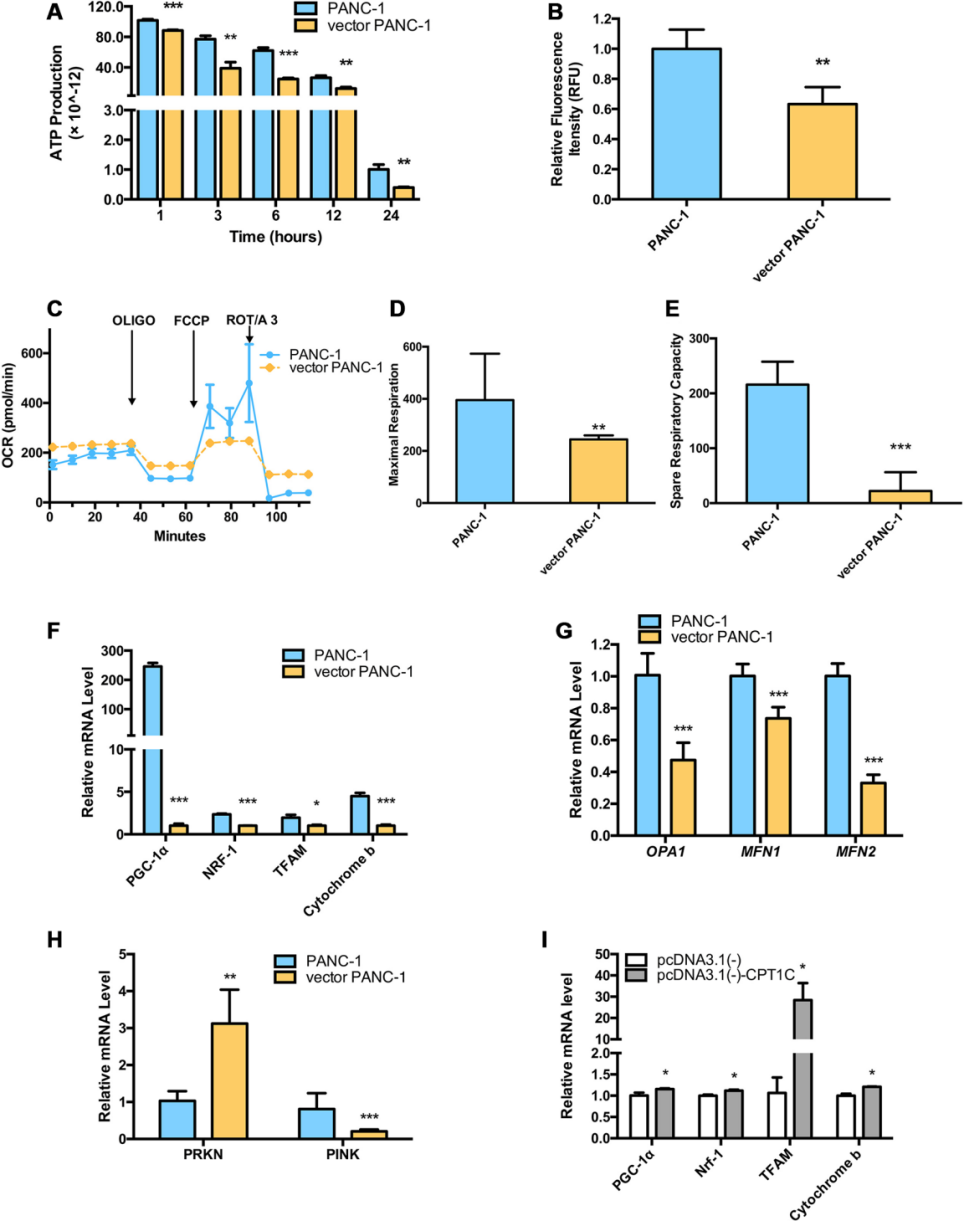
**Dysfunctional mitochondria in low-CPT1C-expressing senescent vector PANC-1 cells.** (**A**) ATP production in senescent vector PANC-1 cells, the magnitude of this difference increased as the time in PBS was extended to 24 h. Data are presented as the mean ± S.E.M, n = 4 (**p < 0.01, ***p < 0.001). (**B**) Loss of mitochondrial transmembrane potential measured by the rh123 dequenching method in senescent vector PANC-1 cells. Data are presented as the mean ± S.E.M, n = 4 (**p < 0.01). (**C**) Mitochondrial integrity in the forms of OCRs (pMol O_2_.min^-1^) in senescent vector PANC-1 cells. Data are presented as the mean ± S.E.M, n = 3. (**D**) Maximal respiration capacity in the form of OCRs in senescent vector PANC-1 cells. Data are presented as the mean ± S.E.M, n = 3 (**p < 0.01). (**E**) Spare respiratory capacity in the form of OCRs in senescent vector PANC-1 cells. Data are presented as the mean ± S.E.M, n = 3 (***p < 0.001). (**F**) Mitochondriogenesis analysis in senescent vector PANC-1 cells. Data are presented as the mean ± S.E.M, n = 3 (*p < 0.05, ***p < 0.001). (**G**) The mitochondrial network structure integrity analysis on the senescent cells. Data are presented as the mean ± S.E.M, n = 3 (***p < 0.001). (**H**) Mitochondrial autophagy analysis on the senescent cells. Data are presented as the mean ± S.E.M, n = 3 (**p < 0.01, ***p < 0.001). (**I**) Mitochondriogenesis analysis on senescent vector PANC-1 cells gaining of CPT1C function. Data are represented as mean ± S.E.M, n = 4 (*p< 0.05). See also Supplementary Figure 4.

CPT1C catalyzes the transportation of fatty acids from the cytoplasm to the mitochondrial matrix for β-oxidation in the mitochondrial outer member. Mitochondrial function was further examined in senescent vector PANC-1 cells. Notably, vector PANC-1 cells produced significantly less ATP under conditions of culture medium deprivation (Figure 3A). Moreover, the reduced intensity of absorbed rh123 dye indicated the loss of mitochondrial transmembrane permeability upon low CPT1C expression in vector PANC-1 cells (Figure 3B).

Oxygen consumption rates (OCRs) from vector PANC-1 cells exhibited lower maximal respiration after the injection of FCCP than mock PANC-1 cells (Figure 3C, 3D). More importantly, the spare respiratory capacity of vector PANC-1 cells was significantly decreased (Figure 3C, 3E), which was indicative of a reduced ability to respond to an increased energy demand. However, vector PANC-1 cells also exhibited higher basal respiratory rates (Figure 3E) and glycolytic function in the forms of extracellular acidification rates (ECARs) (Supplementary Figure 4A), implying that the cells may adjust to mitochondrial respiration to maintain energy supply homeostasis under a long-term stable transfection-induced progressive senescence program [8]. Together, partially impaired mitochondrial respiration integrity upon low CPT1C expression was observed in senescent vector PANC-1 cells.

*PGC-1a* mRNA levels were reduced in vector PANC-1 cells, and its downstream genes, *NRF-1* and *TFAM* mRNAs, were also decreased. *CYTB*, one of the representative mtDNA-encoded subunits [9], was subsequently lowered (Figure 3F).

Genes encoding mitochondrial fission and fusion proteins, including *MFN1*, *MFN2* and *OPA1*, were significantly reduced in senescent vector PANC-1 cells (Figure 3G), suggesting the mitochondrial network structure integrity was impaired in the senescent cells, which might further explain the impaired mitochondrial respiration and mitochondriogenesis pathways. *PRNK*, encoding PARKIN protein, was also reduced, while *PINK1* mRNA expression increased in the senescent cells (Figure 3H). Combined with Figure 3G, these data suggested that mitochondrial autophagy (mitophagy) process might be hampered and a negative feedback might exist.

On the contrary, enhanced cellular bioenergetics pathways (Figure 3I) were observed in the senescent cells overexpressing CPT1C, which suggested mitochondrial function was restored by CPT1C gain-of-function.

**Figure 4:**
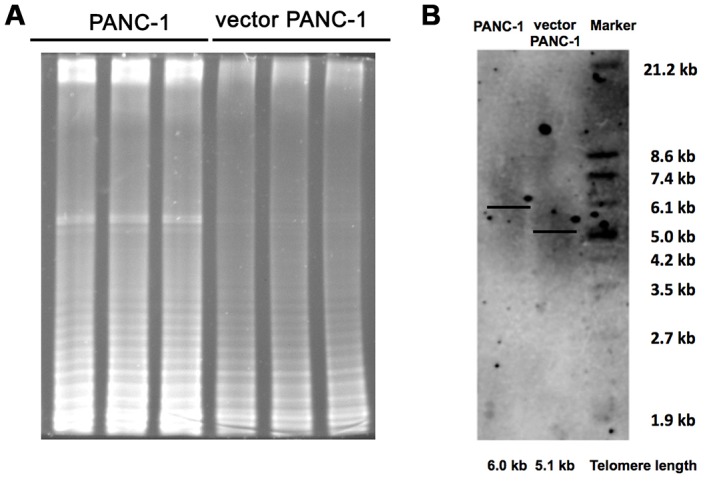
**Inhibited telomere elongation in low-CPT1C-expressing senescent vector PANC-1 cells.** (**A**) Telomerase activity was analyzed with the TRAP assay in mock and vector PANC-1 cells. This experiment was repeated three times. (**B**) Telomere length was determined with the TRF length assay in mock and vector PANC-1 cells.

Additionally, the shortening or structural changes of telomeres at the ends of the chromosomes leads to a DNA damage response and ultimately triggers replicative senescence [10]. Shown as Figure 4A, stable transfection of the vector pCMV remarkably inhibited telomerase activity. As a consequence, the length of the telomere in senescent vector PANC-1 cells shortened by approximately 0.9 kb (Figure 4B).

Taken together, low CPT1C expression contributed to stable transfection-related cell senescence in vector PANC-1 cells, which may result from decreased mitochondrial function and structure integrity, and inhibited telomere elongation in the progressive senescence program. On the contrary, mitochondrial activity was restored by CPT1C gain-of-function in senescent vector PANC-1 cells.

### Malignance are reduced in low-CPT1C-expressing senescent vector PANC-1 cells

**Figure 5:**
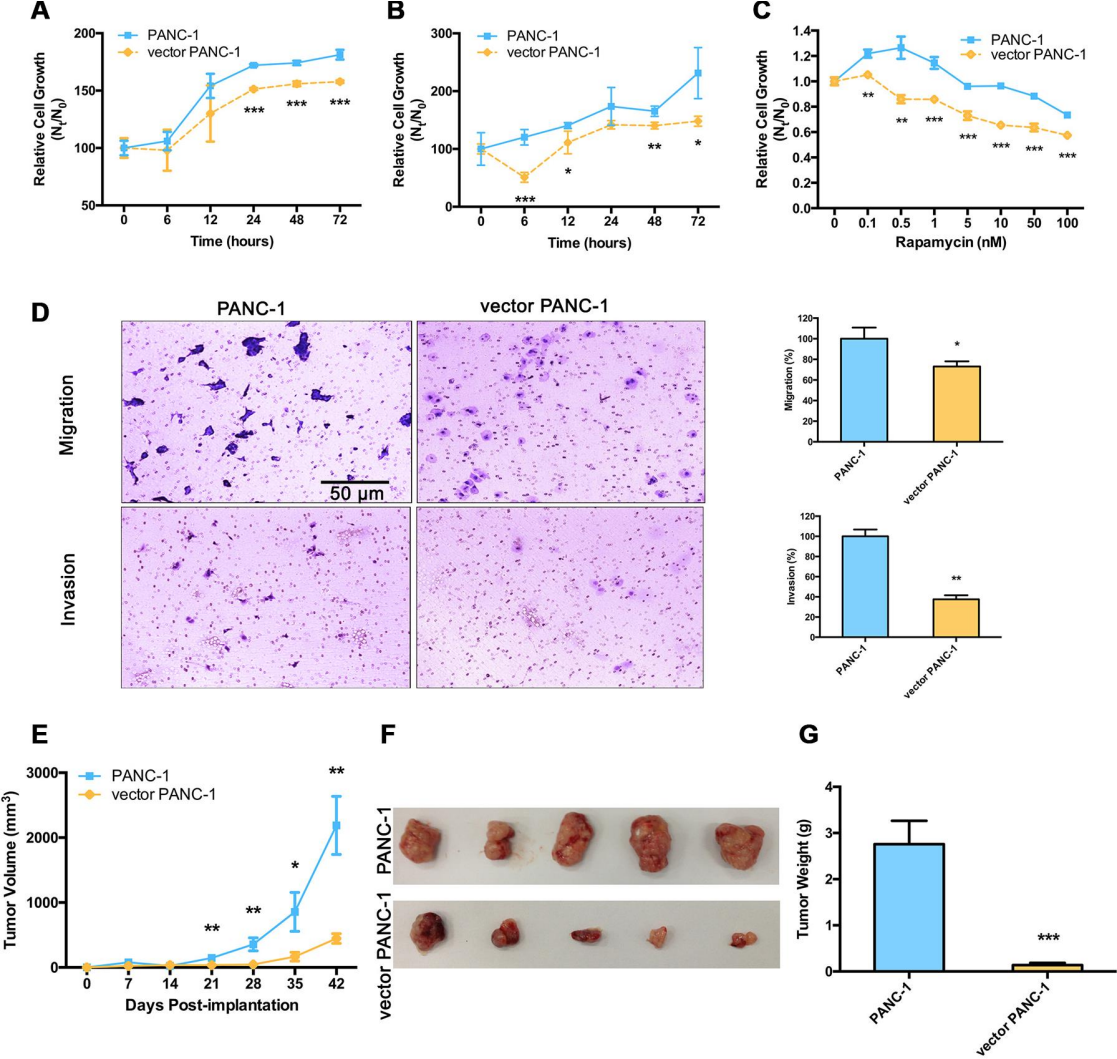
**Malignance is reduced in low-CPT1C-expressing senescent vector PANC-1 cells.** Sensitivity to metabolic stress from (**A**) glucose deprivation (0.5 mM glucose) and (**B**) glycolytic inhibition (20 mM 2-deoxyglucose) of senescent vector PANC-1 cells at the indicated time points. Data are presented as the mean ± S.E.M, n = 5 (*p < 0.05, **p < 0.01, ***p < 0.001). (**C**) Sensitivity to metabolic stress from rapamycin stimuli of senescent vector PANC-1 cells at the indicated concentrations. Data are presented as the mean ± S.E.M, n = 5 (**p < 0.01, ***p < 0.001). (**D**) Transwell migration and Matrigel invasion capacities of senescent vector PANC-1 cells. Data are presented as the mean ± S.E.M, n = 3 (*p < 0.05, **p < 0.01). (**E**) Tumor sizes are presented as the mean ± S.E.M over time, (n = 5) (**p < 0.01, ***p < 0.001). (**F**) Images of tumors after excision on day 42 post-implantation. (**G**) Comparison of dissected tumor weights (mean ± S.E.M, ***p < 0.001). See also Supplementary Figure 5.

The consequences of mitochondrial dysfunction in senescent vector PANC-1 cells with low CPT1C expression were further examined. Cells were grown at gradually decreasing concentrations of glucose or increasing doses of the glycolysis inhibitor, 2-deoxyglucose (2-DG), for 72 h; fewer vector PANC-1 cells were detected in cultures (Supplementary Figure 5A, 5B). Vector PANC-1 cells also showed higher sensitivity to 0.5 mM glucose (Figure 5A), or 20 mM 2-DG (Figure 5B). More importantly, low CPT1C expression sensitized vector PANC-1 cells to rapamycin, which is also a source of stress and cytotoxicity, and triggered a marked decrease in cell survival in vector PANC-1 cells (Figure 5C). These data suggest that low CPT1C expression may hinder senescent vector PANC-1 cell growth under various forms of metabolic stress.

Furthermore, compared with controls, the migration and invasion abilities of vector PANC-1 cells were significantly reduced (Figure 5D), thus further attenuating the incidence of tumor progression arising from these cells. Tumors from the senescent vector PANC-1 group grew much more slowly and were lighter than mock control cells (Figure 5E–5G).

Taken together, low CPT1C expression impaired metabolic adaptation and the malignancy of senescent vector PANC-1 cells *in vitro*, which further hindered the ability of the transformed cells to form tumors *in vivo*.

### Signaling pathways involved in low-CPT1C-expressing senescent vector PANC-1 cells

**Figure 6:**
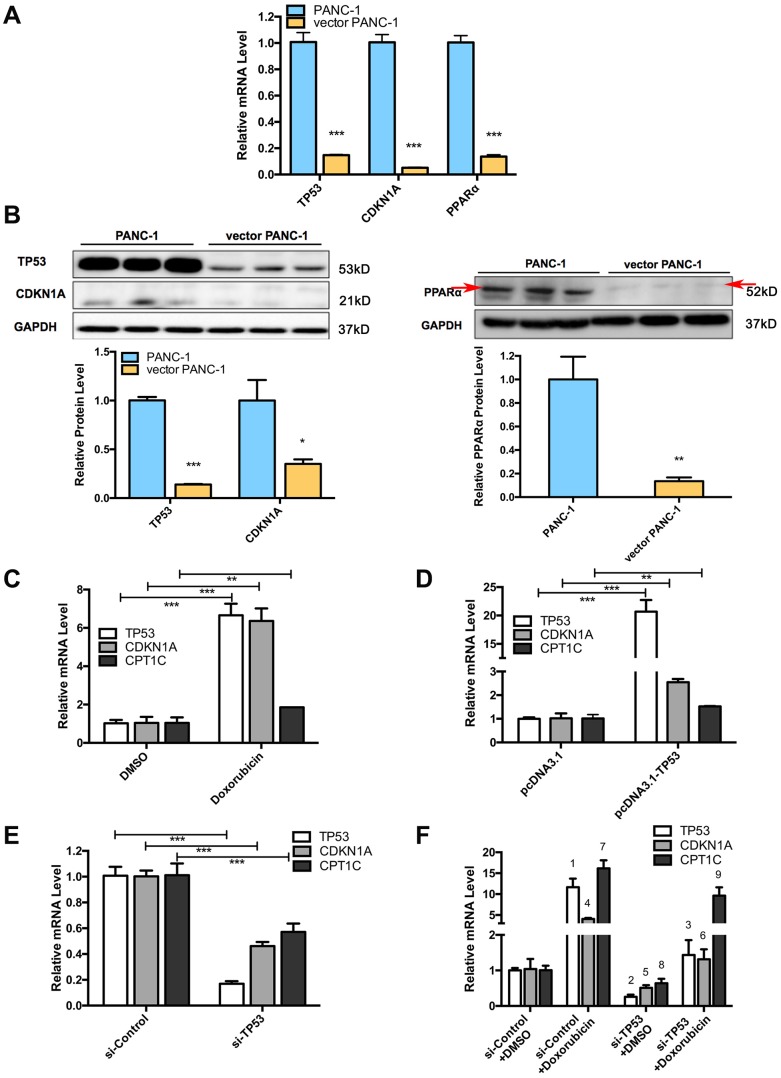
**Signaling pathways involved in low-CPT1C-expression-induced senescence in vector PANC-1 cells and regulation of the TP53 signaling pathway on CPT1C.** (**A**) Quantitative RT-PCR analysis for suppressed genes in senescent vector PANC-1 cells. Data are presented as the mean ± S.E.M, n = 3 (***p < 0.001). (**B**) Images and densitometric analysis for protein bands altered in senescent vector PANC-1 cells. The left panel shared the same GAPDH control with Figure 2D, all these bands were harvested from the same experiment. Data are presented as the mean ± S.E.M, n = 3 (*p < 0.05, **p < 0.01, ***p < 0.001). (**C**) The *CPT1C* mRNA level is upregulated after inducing *TP53* mRNA expression with 0.7 μM doxorubicin (Sigma) for 24 h in PANC-1 cells. (**D**) *CPT1C* mRNA is increased after overexpressing 2 μg of TP53 plasmids for 24 h in PANC-1 cells. (**E**) *CPT1C* mRNA expression was downregulated after knockdown of *TP53* mRNA expression with 50 μM si-TP53 for 72 h in PANC-1 cells. The sequences of specific human siRNAs were commercially available (RiboBio) and listed in Supplementary Table 2. The optimal sense against *TP53* was the following: 5'-GCACAGAGGAAGAGAAUCU dTdT-3'. (**F**) Doxorubicin reversed the si-TP53-induced downregulation of *CPT1C* mRNA expression. For the statistical analysis of *TP53* mRNA expression, ^1^ si-Control+Doxorubicin *vs* si-Control+DMSO, ***p < 0.001; ^2^ si-TP53+DMSO *vs* si-Control+DMSO, ***p < 0.001; and ^3^ si-TP53+Doxorubicin *vs* si-TP53+DMSO, **p < 0.01. For the statistical analysis of *CDKN1A/P53* mRNA level, ^4^ si-Control+Doxorubicin *vs* si-Control+DMSO, ***p < 0.001; ^5^ si-TP53+DMSO *vs* si-Control+DMSO, *p < 0.05; and ^6^ si-TP53+Doxorubicin *vs* si-TP53+DMSO, **p < 0.01. For the statistical analysis of *CPT1C* mRNA expression, ^7^ si-Control+Doxorubicin *vs* si-Control+DMSO, ***p < 0.001; ^8^ si-TP53+DMSO *vs* si-Control+DMSO, *p < 0.05; and ^9^ si-TP53+Doxorubicin *vs* si-TP53+DMSO, **p < 0.01. See also Supplementary Figure 7.

Several crucial signaling components that regulate mitochondrial function and cellular senescence significantly were altered in senescent vector PANC-1 cells. Specifically, a significant decrease in the mRNAs of *PPARα*, *TP53* (*P53*) and its downstream target gene, *CDKN1A* (*P21*), were observed in senescent vector PANC-1 cells (Figure 6A). Reduced protein levels were further verified by immunoblotting (Figure 6B). The decreased *P21* and *P16* (Supplementary Figure 6A) expression levels also suggested the mitochondrial autophagy process was involved in the progressive senescence process [11–13].

The regulation of PPARα on CPT1C was clearly illustrated in our previous study [14]. Here, we further explored how the transcription factor TP53 regulates CPT1C expression. The *CPT1C* mRNA level was upregulated after inducing *TP53* mRNA expression with 0.7 μM doxorubicin (a positive TP53 agonist) for 24 h in PANC-1 cells (Figure 6C). *CPT1C* mRNA also increased after over-expressing 2 μg TP53 plasmids for 24 h in PANC-1 cells (Figure 6D). On the contrary, the *CPT1C* mRNA level was downregulated after knockdown of *TP53* mRNA expression with 50 μM si-TP53 for 72 h (Figure 6E). More importantly, doxorubicin treatment reversed the si-TP53-induced downregulation of *CPT1C* mRNA expression (Figure 6F).

Furthermore, *CPT1C*, a target gene of *PPARα* [14] and *TP53* [15], was identified as a novel biomarker and key regulator of cancer cell senescence through mitochondria-associated metabolic reprogramming [2]. PPARα and TP53/CDKN1A are also crucial signaling components that regulate mitochondrial function and cellular senescence [14, 16]. We further explored the connection between *CPT1C* with *PPARα* and *TP53/CDKN1A* in pancreatic cancer patients (Supplementary Figure 7A). According to data from Collisson Pancreas cohort, *PPARα* mRNA level positively correlated with *CPT1C* mRNA expression. Both *TP53* and *CDKN1A* mRNA levels had no correlation with *CPT1C* mRNA expression in pancreatic cancer patients.

## DISCUSSION

Tumor cell lines are widely used experimental models for determining the molecular events that drive cell growth. However, extended cell culture *in vitro* inevitably triggers culture-related changes of phenotypes, including cellular senescence [17, 18]. In general, cellular senescence can be divided into replicative senescence (RS) and premature senescence. RS has been described for all metabolically active cells that undergo a spontaneous decline in growth rate [19]. Our previous study confirmed that low CPT1C expression plays a crucial role in RS and further identified CPT1C as a novel biomarker and key regulator of cancer cell senescence through mitochondria-associated metabolic reprogramming [2].

Here, PANC-1 cells stably transfected with the empty vector, under antibiotic selection with passaging, fell into a senescence-like growth suppression and cellular senescence process, even without introducing an exogenous gene. Metabolomics profiling indicated reduced acylcarnitines resulting from the decreased expression of their upstream regulatory gene, CPT1C. A decreased CPT1C level caused mitochondrial dysfunction, impaired mitochondrial network structure integrity, and inhibited telomere elongation, further impaired cell survival under metabolic stress, and suppressed the malignance and tumorigenesis of senescent vector PANC-1 cells. On the contrary, mitochondrial activity was restored by CPT1C gain-of-function in the senescent vector PANC-1 cells. Taken together, low CPT1C expression was confirmed to cause mitochondria dysfunction -associated metabolic reprogramming in senescent vector PANC-1 cells. Besides, Lipo2000 reagent was used to establish pCMV stably transfected PANC-1 cell line. The lipid composition of transfection reagent might get into the inner of cells and contribute to the phenotypic changes, such as lipid metabolic reprogramming. Stable transfection manipulation-induced progressive senescence provided further evidence to our previous study that low a CPT1C level plays a crucial role in the mitochondrial dysfunction and metabolic reprogramming-associated RS process [2].

Mounting evidence suggests that aberrant mitochondria trigger cellular senescence [20–22]. In our previous study, mitochondrial dysfunction-mediated senescent phenotypes were observed when CPT1C was efficiently depleted in tumor cell lines. Conversely, CPT1C gain-of-function reversed impaired mitochondrial function and cellular senescence [2]. Deregulation of lipid metabolism was reported to induce senescence process [23], which was further confirmed by our previous study that accumulated lipids and lipid toxicity induced cellular senescence [24]. In the current study, a decreased CPT1C level caused mitochondrial dysfunction and impaired mitochondrial structure integrity on senescent vector PANC-1 cells, further supporting that the absence of CPT1C contributes to mitochondrial dysfunction-related cellular senescence. Down-regulation of mitochondrial fission and fusion genes [25] and the PINK1/PARKIN pathway [26], and the decreased *P21* and *P16* mRNA expression [13, 27], suggested that impaired mitochondrial autophagy process might be induced in senescent cells with low CPT1C expression [28]. On the contrary, mitochondrial activity was restored by CPT1C gain-of-function in senescent cells. Moreover, several well-characterized triggers of senescence were identified, including shortened telomeres, activated oncogenes, and genotoxic and oxidative stress [10, 29]. RS results primarily from the shortening and other structural changes of telomeres at the ends of chromosomes and further triggers the DNA damage response [10]. Here, we demonstrated that stable transfection with the vector pCMV remarkably inhibited telomerase activity and further depressed telomerase activity-catalyzed telomere elongation in senescent vector PANC-1 cells with low CPT1C expression.

The suppression of proliferation of senescent cells, to a large degree, is mediated by cyclin-dependent kinase inhibitor p21^WAF1^ and the tumor suppressor p53 pathway [30, 31]. Depletion of the novel p53 target gene, cpt1c, delays tumor growth in the neurofibromatosis type I tumor model [15]. Hence, we speculated that CPT1C regulates cellular senescence via its upstream gene, TP53. Here, decreased expression of TP53 and its downstream target gene CDKN1A/P21 was observed in senescent vector PANC-1 cells, supporting the crucial role of the TP53/CDKN1A pathway in regulating cellular senescence. Moreover, our previous study demonstrated that PPARα regulates cellular senescence by its novel target gene, CPT1C [14]. Here, PPARα expression was downregulated in senescent PANC-1 cells. In pancreatic cancer patients, the *PPARα* mRNA level positively correlated with *CPT1C* mRNA expression, while both *TP53* and *CDKN1A* mRNA levels had no correlation with *CPT1C* mRNA expression. CDKN1A/P21 is a well-known inhibitor of cell cycle and can arrest the DNA damage-induced cell cycle progression in G1/S and G2/M transitions by inhibiting mitotic CDK4, 6/cyclin-D and CDK2/cyclin-E complexes, respectively [13]. Our data also showed increased G2/M phase arrest in the population of vector PANC-1 cells with senescence phenotypes and low CPT1C expression. TP53-P21 pathway plays a prominent pro-apoptotic role, while reversely it also promotes tumor cell survival in response to metabolic stress [15]. The two-sided effects of TP53-P21 pathway may explain the decrease in TP53, P21 and CPT1C in senescent vector PANC-1 cells. Feedback regulation for cells to maintain homeostasis status in the progressive senescence program, may explain no correlation between *TP53/P21* levels and *CPT1C* expression.

CPT1C, an enzyme located in the outer mitochondrial membrane [32], is involved in lipid metabolism, cellular energy supply [33–35] and other physiological or pathological processes [36–39]. The knockdown of CPT1C inhibited the tumorigenesis of PANC-1 cells *in vivo* [2, 33] and further suppressed xenograft tumor growth *in situ* [2], suggesting that CPT1C represents a therapeutic target for cancer treatment. In summary, stable transfection-induced progressive PANC-1 cell senescence is involved in decreased CPT1C expression through the collapse of mitochondrial function-associated metabolic reprogramming. These results confirm the need to identify cell culture alterations after stable transfection, particularly when used for metabolomics and mitochondria-associated studies, and further suggest that inhibition of CPT1C has the potential to be a target to intervene pancreatic tumorigenesis.

## MATERIALS AND METHODS

### Cell culture

PANC-1 cell line was purchased and authenticated with STR genotyping from Guangzhou Cellcook Biotech Co., Ltd., and further verified without mycoplasma contamination (Beyotime).

Cells were grown in the Dulbecco's modified Eagle's medium (DMEM, Cellgro) with 10% fetal bovine serum (FBS, Gibco), 100 U/mL penicillin sodium (Lonza) and 100 μg/mL streptomycin sulfate (Lonza) at 37 °C in a humidified atmosphere with 5% CO_2_.

### Establishment of pCMV stably transfected PANC-1 cell line

As shown in our previous study [40], 2 × 10^5^ PANC-1 cells in exponential growth were seeded into a well of a 6-well plate. After 24 hours, cells were transfected with 2.5 mg of pCMV empty vector with a selectable marker using Lipo2000 (Invitrogen). Culture media was replaced after 24 hours, then cells were selected with 500 mg/mL G418. After 3~5 weeks, G418-resistant clones were selected with a cloning ring for amplification in further culture.

### Senescence-like growth suppression and cellular senescence analysis

Cell morphology photographs were taken by a microscope (×200, Olympus). Cells plated onto coverslip (NEST) were fixed with 4% paraformaldehyde, cell nuclei were stained with ProLong Gold Antifade Reagent with DAPI (Invitrogen), the cell nuclei morphology was then observed with a laser confocal microscope (Zeiss). Cells fixed in cold 70% ethanol overnight were stained with propidium iodide (PI, Beyotime) staining buffer in the dark at 37 °C for 30 min, cell cycle was then analyzed on an EPICS XL flow cytometer (Beckman Coulter). Cells were labeled with 5-bromo-29-deoxyuridine (BrdU) for 72 h, then fixed *in situ* and incubated with anti-BrdU antibodies for 90 min. Rinsing with PBS, cells were incubated with substrate solution for 5 min. The absorbance was measured at 370 nm with a reference wavelength at 492 nm [41]. A cell growth curve was also drawn to observe the cell growth rate. Cells were seeded into 6-well plates at a density of 1 × 10^5^ cells/well, cultured under standard culture conditions, and counted under a microscope with trypan blue on the indicated days. 5000, 2500 or 1250 cells were cultured for 2 weeks, then fixed and stained with Diff-Quik (Propbs), colony areas were quantified with ImageJ software to investigate colony formation ability. The induction of *IL-8* mRNA expression was analyzed by quantitative RT-PCR to evaluate SASP as previously reported [7]. The SA-β-gal assay (Beyotime) is the most commonly used method to detect senescent cells [42]. Cells were fixed and stained with staining working solution containing X-Gal at 37 °C overnight, the population of SA-β-gal positive cells was then counted.

### Apoptosis pathway analysis

For apoptosis pathway analysis, the *TNF-α*, *TNFR1* and *Fas* mRNA expression was analyzed by quantitative RT-PCR [43].

### Metabolomics analysis

The collection, preparation and UPLC-ESI-QE-MS analysis on the cell sample for metabolomics were performed as described in our previous reports [3, 44]. An aliquot (5 μL) of samples were subjected to an ultra-high performance liquid chromatography electrospray ionization mass spectrometry (UHPLC-ESI-MS). Hydrophilic interaction chromatography (HILIC) separation was performed with an Atlantis Silica HILIC column (3 μm, 2.1 mm i.d. × 100 mm, Waters) on a Thermo Scientific Dionex Ultimate 3000 UHPLC system. MS was performed with a Thermo Scientific Q ExactiveTM benchtop Orbitrap mass spectrometer in ESI positive or negative mode (Thermo Scientific). Untargeted profiling analysis acquired the data followed by Top-10 data-dependent MS/MS. Total ion chromatograms and mass spectra from LC-HRMS runs were generated as raw files in Xcalibur (Thermo Scientific).

Optimization for comprehensive metabolite phenotypes between two groups and discovering differential metabolites, data mining consisting of background noise subtraction, automated peak detection and integration, peak alignment, multivariate principal component analysis (PCA) and univariate analysis, were performed with Thermo Scientific label-free differential analysis bioinformatics software SIEVE 2.2 (Thermo Scientific) and SIMCA-P 13.0 (Umetrics). Subsequently, metabolites were putatively identified based on accurate mass match and fragmentation pattern match. With the mass-to-charge (m/z) ratio of the metabolic features, putative structural annotation was carried out by retrieving the metabolite databases HMDB (http://www.hmdb.ca/), KEGG (http://www.genome.jp/kegg/) and METLIN (http://metlin.scripps.edu). Mz Cloud (https://www.mzcloud.org/) and METLIN were used to match MS/MS spectral.

### Quantitative RT-PCR analysis

Analysis was performed as described in a previous report [45]. Cells were lysed with Trizol (Invitrogen), and total RNA was extracted with ethanol precipitation. RNA concentration was measured with a NanoDrop Flex Station 3 (Molecular Device). 1 μg purified RNA was randomly reverse-transcribed to cDNA with the PrimeScript RT reagent kit with gDNA eraser (TaKaRa Biotech). Real-time PCR was then performed with SYBR Premix Ex Taq II (Tli RnaseH Plus) kit (TaKaRa Biotech) in a 7500 Real-Time PCR System (Applied Biosystems). Raw gene expression values were normalized with housekeeping *ACTB* mRNA from the same reaction. The specific human primers obtained from website of http://pga.mgh.harvard.edu/primerbank/ and PrimerDepot, were commercially available at Invitrogen Corporation and shown in Supplementary Table 1.

### Western blot analysis

Analysis was performed as described in a previous report [46]. Cells were homogenized with the Radioimmunoprecipitation assay (RIPA) lysis buffer containing phenylmethylsulfonyl fluoride (PMSF). Concentrations of prepared proteins were determined with the BCA protein assay (Thermo Scientific). Proteins were electrophoresed with a 10% SDS-PAGE and transferred to the Polyvinylidene Fluoride (PVDF) membranes (Millipore). 5% nonfat milk in Tris-Buffered-Saline with Tween (TBST) was used to block the nonspecific banding, then PVDF membranes were incubated with primary antibody overnight at 4°C. The antibodies used were: CPT1C (Cat. No.: #ab87498, Abcam), TP53 (#ab26, Abcam), P21 (#sc-271610, Santa Cruz), PPARα (#ab24509, Abcam) and GAPDH (#2118, Cell Signaling Technology). The immunoblot bands were visualized by anti-rabbit horseradish peroxidase-linked secondary antibody (Cell Signaling Technology) for 1 h at 37°C. After washing with TBST for 3 times, protein-antibody complexes were detected with the Electrochemiluminescence (ECL) detection kit (Engreen Biosystem) on a chemiluminescence imaging system (GE Healthcare). The intensity of immunoblot bands was quantitated with the Quantity One software (Bio-Rad Laboratories).

### Mitochondrial dysfunction analysis

Analysis was performed as described in our previous study [2]. To measure the total ATP production, culture medium was replaced with PBS for 12 h and luminescence representing cellular ATP production levels was detected with the CellTiter-Glo Luminescent Cell Viability Assay (Promega) on a FlexStation 3 (Molecular Devices). The mitochondrial membrane depolarization was measured via fluorescence dequenching of 0.4 μM rh123 (Sigma) as previously reported [47]. The mean rh123 (excitation: 485 nm, emission: 535 nm) fluorescent intensity of 25 fields in each well was calculated with ArrayScanVTI High Content Application (Thermo Fisher). Cells were seeded into XF24 V7 cell culture plates (Seahorse Bioscience) with the same cell density to analyze mitochondrial respirations between the two groups as previously described [48]. The culture medium was replaced with XF assay medium (Seahorse Bioscience) on the next day. After incubation without CO_2_ for 1 h, the rate of change for dissolved O2 concentration (measurement of OCR) was measured by an XF24 Extracellular Flux Analyzer (Seahorse Bioscience). Cells were incubated under four conditions sequentially: (a) basal respiration was measured without additives; (b) 1 μM oligomycin was added to inhibit ATP synthase, mitochondrial Complex V and oxidative phosphorylation (OXPHOS); (c) the maximal mitochondrial respiration was induced by adding 1 μM carbonyl cyanide-4-trifluoro methoxy phenyl hydrazine (FCCP, a mitochondrial uncoupler); and (d) the reaction was ended with 1 μM rotenone and 1 μM antimycin A (mitochondrial Complex I and III inhibitors and poisons). Considering the size of senescent vector PANC-1 cells was larger than the untreated PANC-1 cells, the mitochondrial respiration OCRs were normalized with the total protein concentration of each group after harvesting and lysing the cells for the Seahorse experiment. Mitochondrial respirations coupled to ATP synthesis were obtained with subtracting oligomycin responses from the basal OCRs. The difference between maximal respirations and basal respirations was defined as spare respiratory capacity. Mitochondriogenic pathways and mitochondrial-encoded mRNAs were measured via quantitative RT-PCR analysis as previously described. For analysis of the integrity of the mitochondrial network, the *MFN1*, *MFN2* and *OPA1* levels were measured by quantitative RT-PCR [25]. Mitophagy was analyzed for the *MFN1*, *MFN2* and *OPA1* levels and PINK1/PARKIN pathway using quantitative RT-PCR [26].

### Telomerase activity and telomere length assays

Telomerase activity analysis was performed with a telomeric repeat amplification protocol (TRAP) assay. Briefly, approximately 2 × 10^6^ cells were resuspended in lysis reagent. Telomeric DNA was elongated and purified, and amplification was performed by PCR. PCR products were separated on a 10% nondenaturing PAGE gel. The gel was fixed, stained using Gel-red (Biotium) and photographed. The terminal restriction fragment (TRF) length assay was performed to determine the telomere length as described in a previous report [41, 49]. Genomic DNA was digested with Hinf1/Rsal restriction enzymes. The digested DNA fragments were electrophoresed on a 0.8% agarose gel, transferred to a nylon membrane by capillary transfer and then fixed on a wet blotting membrane by UV crosslinking. The membrane was hybridized with a DIG-labeled hybridization probe for telomeric repeats and incubated with anti-DIG-alkaline phosphatase. The TRF was determined by chemiluminescence detection (GE Healthcare).

### Metabolic stress stimuli

Analysis was performed as previously described [50]. The cells were incubated in culture medium deprived of glucose (Sigma). 2-DG (Sigma) in PBS was diluted to the required concentrations with culture medium. Cells were further treated with pre-optimized 0.5 mM glucose or 20 mM 2-DG at the indicated days. Rapamycin (Chemietek) in DMSO was diluted to the indicated concentrations with culture media, SRB staining was performed to evaluate the cell growth after 72 h.

### Sulforhodamine B (SRB) assays

Analysis was performed as previously described [51]. Cells were fixed *in situ* with 50 μL of pre-cold 50% (w/v) trichloroacetic acid (TCA) for 1 h at 4°C. After washing with ultrapure water and drying, each well was incubated in 100 μL of 0.4% SRB solution (w/v, SRB dissolved in 1% (v/v) acetic acid) for 30 min at room temperature. After washing with 1% acetic acid and drying, SRB stains were solubilized in 10 mM Tris-HCl (pH=10.5) and absorbance was determined at 515 nm.

### Transwell migration and matrigel invasion assays

Assays were employed as described in our previous reports [3, 50]. Transwell migration and matrigel invasion assays were employed to evaluate cell migration and invasion capacities, respectively. Briefly, cells suspension of 3×10^4^ was added to cell culture inserts (Corning) containing a polycarbonate filter with 8 μm diameter pores blocked with 2.5% BSA for transwell migration assay or coated with 1:5 dilution matrigel 100 ul/well for matrigel invasion assay. Cells were incubated for 17 h under standard culture conditions. Tumor cells remaining on the topside of the membrane or gel were removed, and cells that had migrated or invaded to the underside were fixed and stained with Diff-Quik (PolySciences). Five fields per insert were photographed and the number of cells was counted under microscope.

### Xenograft mice assays

Mouse xenograft model were used as previously described [3]. 6~8 weeks old athymic male nude mice (BALB/c-nu/nu) were purchased from the Laboratory Animal Center of Sun Yat-sen University, and maintained with water and chow provided *ad libitum* under standard 12 h light/ 12 h dark cycle. Exponentially-growing cells were injected subcutaneously into the right flank of mice (5 × 10^6^ cells in 0.2 ml of 50% matrigel basement membrane matrix (BD Biosciences) per mouse). Tumor sizes and body weights were measured once a week for 6 weeks, tumor length and width were measured with a digital caliper. The formula utilized to calculate was: tumor volume (mm^3^) = 3.14/6*((tumor length + width)/2)^3. Mice were sacrificed when the tumors reached 2 cm in diameter, and xenograft tumors were extracted and weighed.

### Bioinformatics analysis

All cohort data were downloaded at https://www.oncomine.org [52] and http://www.cbioportal.org [53, 54]. The *CPT1C* reporter (probe) is 227468_at, the *PPARα* reporter is 206870_at, the *TP53* reporter is 201746_at, the *CDKN1A* reporter is 1555186_at, the *IL-8* reporter is 202859_x_at in Collisson Pancreas cohort (n = 27).

### Plasmid transit transfection

Analysis was performed as described in a previous report [55]. *Human* CPT1C expression plasmid was commercially constructed by Genechem Corporation. *Human* TP53 expression plasmid was obtained from Addgene nonprofit plasmid repository. Briefly, the cells were grown in 6-well plates at 80% confluence then transfected with plasmids using Lipo2000 reagent (Invitrogen) in antibiotic-free culture medium following the instruction of manufacturer. More detailed, 4 μL Lipo2000 reagent or 2 μg DNA was diluted well in 250 μL Opti-MEM medium then incubated for 5 mins respectively. 250 μL diluted DNA was added to 250 μL diluted Lipo2000 reagent (1:1 ratio) then incubated for 20 mins. 500 μL DNA-reagent complex was added to the culture medium of each well and mixed gently. Quantitative RT-PCR was performed at 24 h post-transfection as described above.

### Gene silencing by RNA interference

Silencing with siRNA was performed as previously described [56]. Cells were seeded into 6-well plates at 1 × 10^5^ cells/well. Cells were transfected with 50 nM siRNA or siControl using the Lipofectamine RNAiMAX (Life Technologies) on the second day. Quantitative RT-PCR was performed at 72 h post-transfection as described above. The sequences of specific *human* siRNAs were commercially available (RiboBio) and listed in Supplementary Table 2.

### Statistical analysis

All values were expressed as the mean ± S.E.M. Two-tailed Student’s t tests and graphs were performed using GraphPad Prism v6.0c software. Significance is represented by *p < 0.05, **p < 0.01, **p < 0.001 versus control.

## Supplementary Material

Supplementary Figures

Supplementary Tables
