# Supplementary material for: Carnitine palmitoyltransferase 1C contributes to progressive cellular senescence
Source: Aging (Albany NY). 2020 Apr 14;12(8):6733–55. doi: 10.18632/aging.103033 (PMC7202531; doi:10.18632/aging.103033)
Supplement: Supplementary Figures [file aging-12-103033-s002..pdf]

SUPPLEMENTARY FIGURES

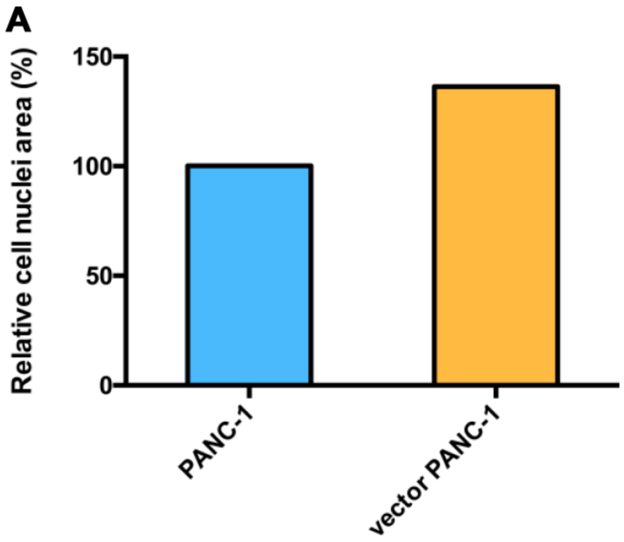

Supplementary Figure 1. (related to Figure 1). Quantification of the cell nuclei size. (**A**) The cell nuclei size was quantified with Image J software.

.

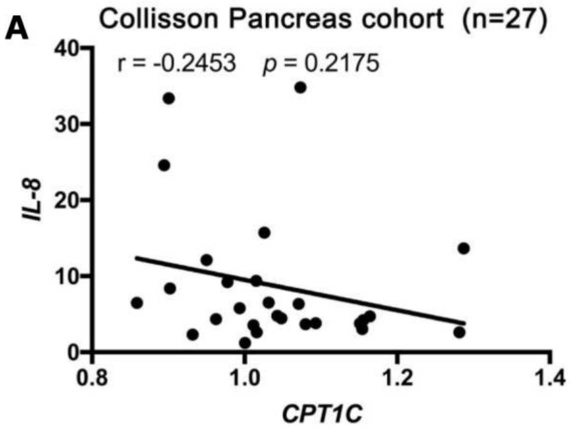

Supplementary Figure 2. (related to Figure 1). Correlation of key SASP factor, IL-8 with CPT1C expression in pancreatic cancer patients. (**A**) *IL-8* negatively correlates with *CPT1C* mRNA expression in pancreatic cancer patients.

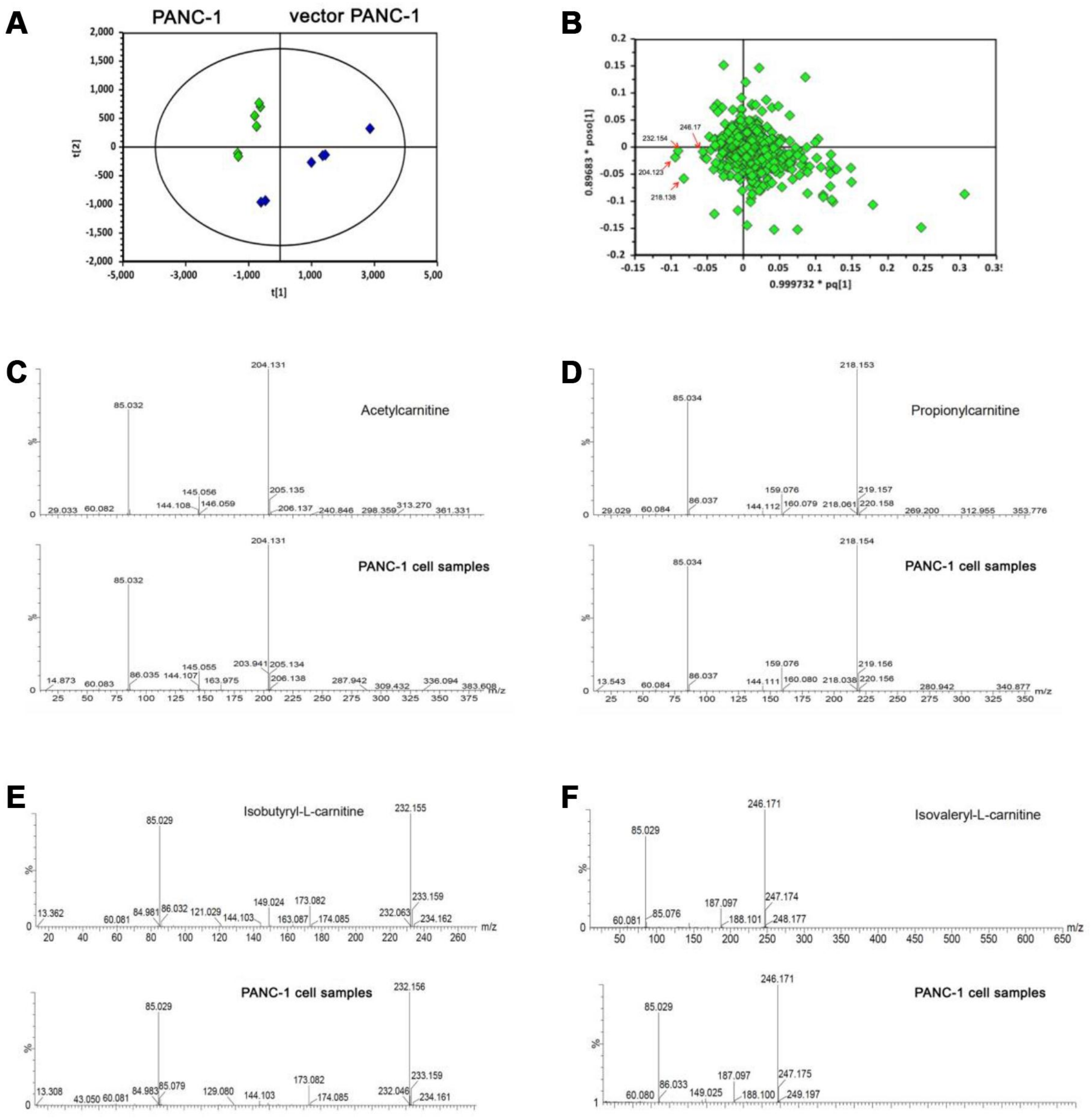

Supplementary Figure 3. (related to Figure 2). Metabolomics profiles reveal significantly decreased acylcarnitines in senescent vector PANC-1 cells. (**A**) PCA score plots of HILIC-ESI$^-$-MS metabolomics profiles obtained from HILIC-ESI$^-$-MS, n = 6/group. (**B**) S-plot of OPLS/DA models of HILIC-ESI$^+$-MS data. (**C**–**F**) Validation of cell metabolite biomarkers by LC-MS/MS analysis. Corresponding MS/MS spectra of authentic chemicals (up) and cell samples (bottom) are shown.

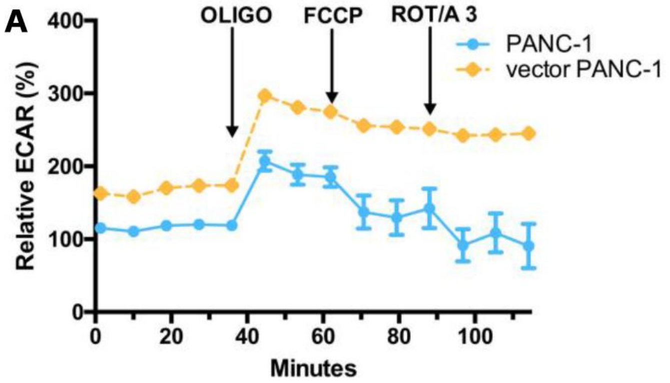

Supplementary Figure 4. (related to Figure 3). Vector PANC-1 cells exhibited higher glycolytic function. (**A**) Glycolytic function in the forms of ECARs (mpH.min^{-1}) in senescent vector PANC-1 cells. Data are presented as the mean ± S.E.M, n = 3.

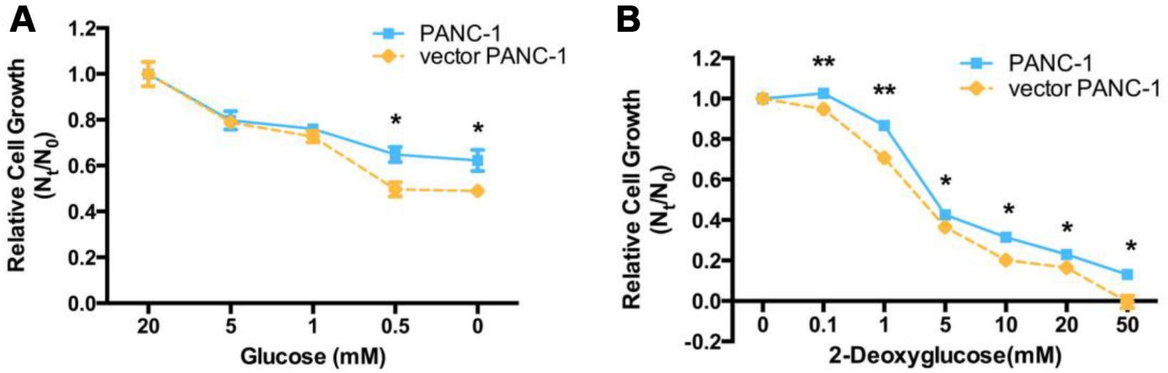

Supplementary Figure 5. (related to Figure 5). Cell sensitivity to metabolic stress. Cell sensitivity to metabolic stress from (**A**) glucose withdrawal and (**B**) glycolytic inhibition in senescent vector PANC-1 cells at the indicated concentrations is shown. Data are presented as the mean ± S.E.M, n = 5 (*$p < 0.05$, **$p < 0.01$).

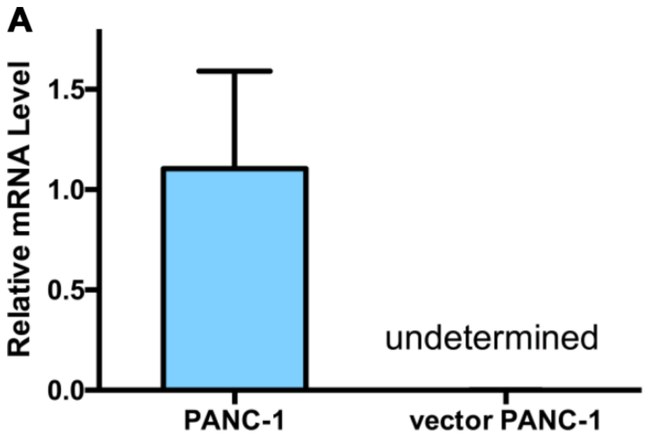

Supplementary Figure 6. Reduced *CDKN1A* (*P16*) mRNA in the senescent vector PANC-1 cells. (**A**) Quantitative RT-PCR analysis for *P16* was performed in the senescent vector PANC-1 cells.

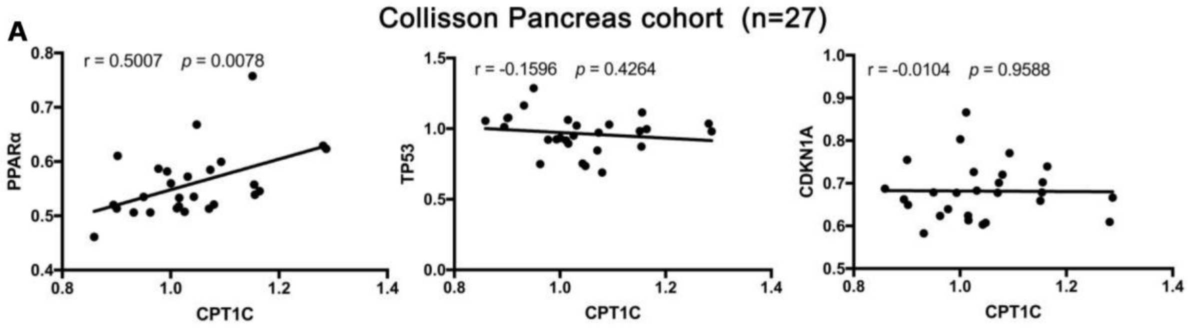

Supplementary Figure 7. (related to Figure 6). Correlation of crucial signaling components of cellular senescence with CPT1C expression in pancreatic cancer patients. (**A**) Correlation analysis between *PPARα*, *TP53*, and *CDKN1A* with *CPT1C* mRNA expression in pancreatic cancer patients (data from the Collisson Pancreas cohort, n = 27).
