# Supplementary material for: Carnitine palmitoyltransferase 1C contributes to progressive cellular senescence
Source: Aging (Albany NY). 2020 Apr 14;12(8):6733–55. doi: 10.18632/aging.103033 (PMC7202531; doi:10.18632/aging.103033)
Supplement: Supplementary Tables [file aging-12-103033-s001..pdf]

SUPPLEMENTARY TABLES

Supplementary Table 1. Sequence of primers for quantitative RT-PCR analysis.

Gene Name	Genebank Accession	Species Specificity	Sequences of Primers
β-actin	NM_001101	Human	forward 5′- CCTTGCACATGCCGGAG-3′<br>reverse 5′-GCACAGAGCCTCGCCTT-3′
CPT1A	NM_001876	Human	forward 5′-GCCTCGTATGTGAGGCAAAA-3′<br>reverse 5′-TCATCAAGAAATGTCGCACG-3′
CPT1B	NM_004377	Human	forward 5′-GGTCCAGTTTACGGCGATAC-3′<br>reverse 5′-CCTCTCATGGTGAACAGCAA-3′
CPT1C	NM_001136052	Human	forward 5′-GGATGGCACTGAAGAGGAAA-3′<br>reverse 5′-TCCTGGAAAAGGCATCTCTC-3′
CPT2	NM_000098	Human	forward 5′-CGGAGTCTCGAGCAGATAGG-3′<br>reverse 5′- GGAAAAGAACTGCATGAGCA-3′
CRAT	NM_001257363	Human	forward 5′-GGGCTCGAGTAGATGACCAC-3′<br>reverse 5′-ATGAGTTTCAGGCCTCAGGA-3′
IL-8	NM_000584	Human	forward 5′- ACTGAGAGTGATTGAGAGTGGAC-3′<br>reverse 5′-AACCCTCTGCACCCAGTTTTC-3′
PGC-1α	NM_013261	Human	forward 5′-AATCCGTCTTCATCCACAGG-3′<br>reverse 5′-GGTGCAGTGACCAATCAGAA-3′
NRF1	NM_001040110	Human	forward 5′-AGGAACACGGAGTGACCCAA-3′<br>reverse 5′-TATGCTCGGTGTAAGTAGCCA-3′
TFAM	NM_003201	Human	forward 5′- ATGGCGTTTCTCCGAAGCAT-3′<br>reverse 5′-TCCGCCCTATAAGCATCTTGA-3′
Cytochrome b	NM_000101	Human	forward 5′-CCCAGTGGTACTTTGGTGCC-3′<br>reverse 5′-GCGGTCATGTACTTCTGTCCC-3′
TP53	NM_001126118	Human	forward 5'-CAGCACATGACGGAGGTTGT-3'<br>reverse 5'-TCATCCAAATACTCCACACGC-3'
CDKN1A	NM_078467	Human	forward 5'-TGTCCGTCAGAACCCATGC-3'<br>reverse 5'-AAAGTCGAAGTTCCATCGCTC-3'
PPARα	NM_005036	Human	forward 5'-CGGTGACTTATCCTGTGGTCC-3'<br>reverse 5'-CCGCAGATTCTACATTCGATGTT-3'
TNFA	NM_000594	Human	forward 5'-GAGGCCAAGCCCTGGTATG-3'<br>reverse 5'-CGGGCCGATTGATCTCAGC-3'
TNFR1	NM_001065	Human	forward 5'-AACGAGTGTGTCTCCTGTAGT-3'<br>reverse 5'-GGAGTAGAGCTTGGACTTCCAC-3'
FAS	NM_000043	Human	forward 5'-AGATTGTGTGATGAAGGACATGG-3'<br>reverse 5'-TGTTGCTGGTGAGTGTGCATT-3'
FASL	NM_000639	Human	forward 5'-TGCCTTGGTAGGATTGGGC-3'<br>reverse 5'-GCTGGTAGACTCTCGGAGTTC-3'
PRKN	NM_004562	Human	forward 5'-CCCACCTCTGACAAGGAAACA-3'<br>reverse 5'-TCGTGAACAAACTGCCGATCA-3'
PINK1	NM_032409	Human	forward 5'-GGAGGAGTATCTGATAGGGCAG-3'<br>reverse 5'-AACCCGGTGCTCTTTGTCAC-3'
CDKN2A	NM_000077	Human	forward 5'-ATGGAGCCTTCGGCTGACT-3'<br>reverse 5'-GTAACTATTCGGTGCGTTGGG-3'
OPA1	NM_015560	Human	forward 5'-CTGCAGGTCCCAAATTGGTT-3'<br>reverse 5'-TCTTTGTCTGACACCTTCCTGT-3'
MFN1	NM_033540	Human	forward 5'-CGGGGTGACCTTCGAGC-3'<br>reverse 5'-TTCTGCCATTATGCACCTGGA-3'
MFN2	NM_001127660	Human	forward 5'-AGCGTTCAGAGGCCATCG-3'<br>reverse 5'-TCCAAGCTTCTTCACCTTCCC-3'

Supplementary Table 2. Sequences of RNAi assay.

RNAi Name	Species Specificity	Sequences
siRNA TP53-1	Human	5′-GGACAUACCAGCUUAGAUU dTdT-3′
siRNA TP53-2	Human	5′-GCACAGAGGAAGAGAAUCU dTdT-3′
siRNA TP53-3	Human	5′-GACUCCAGUGGUAAUCUAC dTdT-3′
